# Drive soil nitrogen transformation and improve crop nitrogen absorption and utilization - a review of green manure applications

**DOI:** 10.3389/fpls.2023.1305600

**Published:** 2024-01-04

**Authors:** Hanqiang Lyu, Yue Li, Yulong Wang, Pengfei Wang, Yongpan Shang, Xuehui Yang, Feng Wang, Aizhong Yu

**Affiliations:** ^1^ College of Agronomy, Gansu Agricultural University, Lanzhou, Gansu, China; ^2^ State Key Laboratory of Aridland Crop Science, Gansu Agricultural University, Lanzhou, Gansu, China

**Keywords:** green manure application, nitrogen management, immobilization, mineralization, soil -plant system

## Abstract

Green manure application presents a valuable strategy for enhancing soil fertility and promoting ecological sustainability. By leveraging green manures for effective nitrogen management in agricultural fields can significantly reduce the dependency of primary crops on chemical nitrogen fertilizers, thereby fostering resource efficiency. This review examines the current advancements in the green manure industry, focusing on the modulation of nitrogen transformation in soil and how crops absorb and utilize nitrogen after green manure application. Initially, the influence of green manure on soil nitrogen transformation is delineated, covering processes such as soil nitrogen immobilization, and mineralization, and losses including NH_3_, N_2_O, and NO_3_^−^-N leaching. The review then delves into the effects of green manure on the composition and function of soil microbial communities, highlighting their role in nitrogen transformation. It emphasizes the available nitrogen content in the soil, this article discussing nitrogen uptake and utilization by plants, including aspects such as nitrogen translocation, distribution, the root system, and the rhizosphere environment of primary crops. This provides insights into the mechanisms that enhance nitrogen uptake and utilization when green manures are reintroduced into fields. Finally, the review anticipates future research directions in modulating soil nitrogen dynamics and crop nitrogen uptake through green manure application, aiming to advance research and the development of the green manure sector.

## 1 Introduction

For a substantial period preceding the widespread use of synthetic nitrogen fertilizers, green manure played a pivotal role in global food security and agricultural development (Meena et al., 2018). Even in contemporary agriculture, green manure continues to exert significant effects on soil enhancement, fertilizer substitution, and ecological protection (Spiertz, 2009). China pioneered the green manure production system before the 3rd century AD, utilizing leguminous green manure, crop rotation, and intercropping to create diverse cropping structures for fertilizing farmland (Liu et al., 2013). By the early 19th century, the practice spread from China to Europe and the Americas, evolving into cover crops. Advancements in modern science and technology have gradually unveiled the mechanisms underlying improving farmland productivity and ecological services through long-term green manure cultivation and incorporation (Fageria, 2007). Current research indicates that, as an external source of organic matter, long-term green manure incorporation primarily manifests ecological effects through improvements in soil physicochemical properties, such as aggregates and organic matter, and enhancements in biological functions, represented by microorganisms (Abbott et al., 2018; Bungau et al., 2021). Additionally, due to its intrinsic characteristics, green manure actively participates in water and nutrient regulation processes in crop-soil systems post-incorporation, contributing to energy conservation, increased yield and quality, and enhanced water and nutrient use efficiency (Zhang et al., 2010). Moreover, long-term green manure cultivation plays a positive role in preventing soil erosion and inhibiting weeds and pests in farmland (Chimouriya et al., 2018; Maitra et al., 2018).

The extended use of chemical fertilizers and continuous cropping patterns contribute to soil quality degradation, structural disequilibrium, and reduced efficiency in nitrogen absorption and utilization by crops (Bai et al., 2015; He et al., 2018; Li P. et al., 2021). Furthermore, non-point source pollution from agricultural activities, exacerbated by fertilizer applications, is evolving into an agricultural ecological predicament (Ju and Zhang, 2017). Concurrent with advancements in agricultural techniques and the promotion of sustainable development ideologies, transformative shifts are evident in global agricultural practices. Green initiatives are positioned to shape the trajectory of future agricultural endeavors (Horlings and Marsden, 2011). As a vital element of this green paradigm, green manures are gaining prominence in the roadmap for the upcoming agricultural era. This emphasis centers around adjusting crop structures, mitigating non-point source pollution, rejuvenating farmland ecosystems, integrating land cultivation with fertilization strategies, and enhancing the quality and efficacy of agricultural yields (Rees and Chow, 2005). Historically, green manure crops have been utilized as catch crops to expedite nitrogen turnover within farmland ecosystems, reinforcing the consistency and augmentation of grain outputs (Zhang et al., 2015; Zhang et al., 2016). Notably, leguminous green manure, recognized as a comprehensive bio-organic fertilizer, facilitates biological nitrogen fixation, asserting its indispensable influence in soil enrichment and moisture conservation (Kim et al., 2012). In the realm of nitrogen dynamics, green manure contributes to humus formation, mineralization, ammonification, nitrification, denitrification, and the assimilation and sequestration of accessible nitrogen by relevant functional microorganisms (Mancinelli et al., 2013). Simultaneously, the incorporation of green manures can modulate the nitrogen assimilation and deployment within principal crops by engaging in nitrogen fixation and mineralization processes (Martens and Entz, 2011; Momesso et al., 2022).

**Figure 1:**
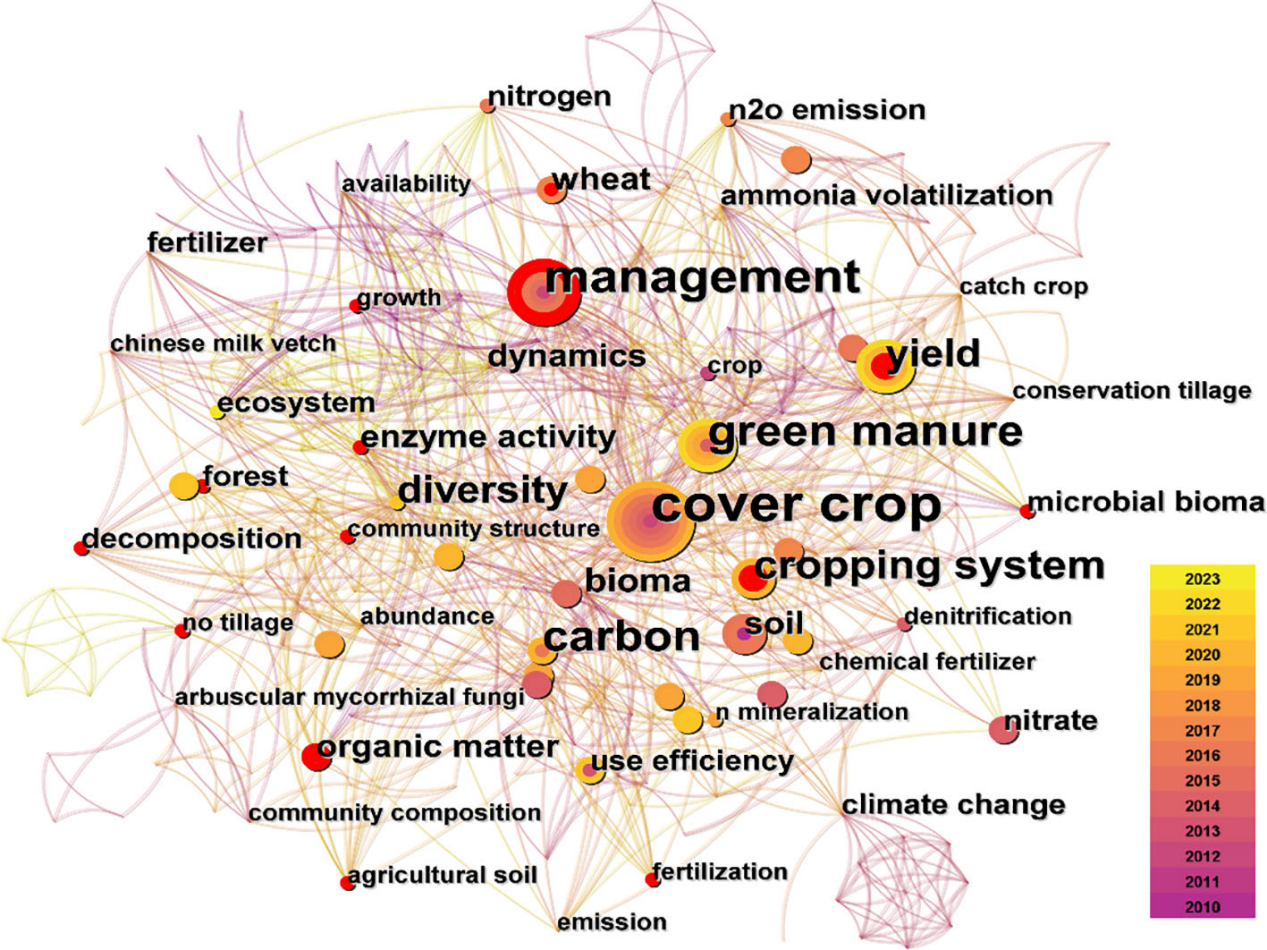
The research hotspots of green manure application in 2010 – 2023.

Numerous studies have explored the influence of green manure application on nitrogen absorption and utilization in cereal crops, such as wheat (*Triticum aestivum*), corn (*Zea mays*), potatoes (*Solanum tuberosum*), and rice (*Oryza sativa*) (see **Figure 1** and **Table 1**). While these empirical findings contribute significantly to bridging theoretical gaps in the field of green manure application, there is a lack of summarized and evaluated conclusions to provide references for subsequent researchers. Drawing upon prior research, this review encapsulates the modulatory effects of incorporating green manure on key soil nitrogen transformation processes, including nitrogen fixation, mineralization, loss, crop uptake, and the role of soil microorganisms. Additionally, the study delves into nitrogen transportation and distribution within the crop root system under green manure application, elucidating the mechanisms through which green manure enhances nitrogen utilization in primary crops. This review aims to establish a foundation for advancing research on the ecological service functions associated with green manure application.

**Table 1:** In some countries where green manure crops are widely planted and the ecological benefits produced by green manure applications.

Green manure crops	Planting pattern	Country/Region	Research field	Main benefits	References
Clover (Oxalis)	Rotation	Denmark	Ecological effect	Mitigating weed problems in organic annual crops	(Melander et al., 2020)
Hairy vetch (*Vicia villosa*); Yellow sweet clover (*Melilotus officinalis*)	Rotation	Norway; Ontario, Canada	Suitability of green manure crops; Economic effectiveness	Hairy vetch was rich in N and showed a high N mineralization rate; The suitability of green manure crops depends on the cultivar, sowing time, and climate region; Increase grain yield and soil nitrogen storage	(Brandsaeter et al., 2008; Yang L. et al., 2019)
Sudangrass (*Sorghum sudanese*); Cowpea (*Vigna unguiculata*); Rye (*Secale cereale*); Pea (*Pisum sativum*)	Rotation	Michigan, USA; Louisiana, USA	Maximum benefits of green manure crops Rotation; No-till and cover crops management practices	Legume green manure crops are the most reliable means to enhance cash crop yields; If soil pests are a major yield-limiting factor in cash crop production, then the use of brassica green manure should be considered	(Snapp et al., 2005; Naveen et al., 2020)
Barley (*Hordeum vulgare*); Oat (*Avena sativa*); Oilseed rape (*Brassica rapa* var.*oleifera*); Faba bean (*Vicia faba*); Lupin (*Lupinus micranthus*); Alfalfa (*Medicago sativ*)	Rotation	Sweden; Germany	Evaluating effects of introducing green manure into crop rotations; Nitrogen leaching; Net greenhouse gas balance and crop productivity	Cropping systems with legumes reduced N_2_O emissions with comparable or slightly lower nitrate-N leaching and had positive phytosanitary effects	(Reckling et al., 2016)
Chickpea (*Cicer arietinum*)	Mono- culture	Algeria	The ability of fixing nitrogen	The nitrogen-fixing nodule function, germinability, and possibly photosynthesis, are revealed	(Souad Insaf et al., 2021)
February orchid (*Orychophragmus violaceus*); Common vetch (*Vicia sativa*); Red bean grass (*Onobrychis viciifolia Scop*)	Rotation	China	Nitrogen replacement technology, N fixation and emission reduction; Soil carbon and nitrogen sequestration	Green manure and chemical fertilizers is an efficient management approach for improving maize yields and NUE simultaneously	(Yang L. et al., 2019)

## 2 Response and key mechanisms of soil nitrogen transformation to green manure application

**Figure 2:**
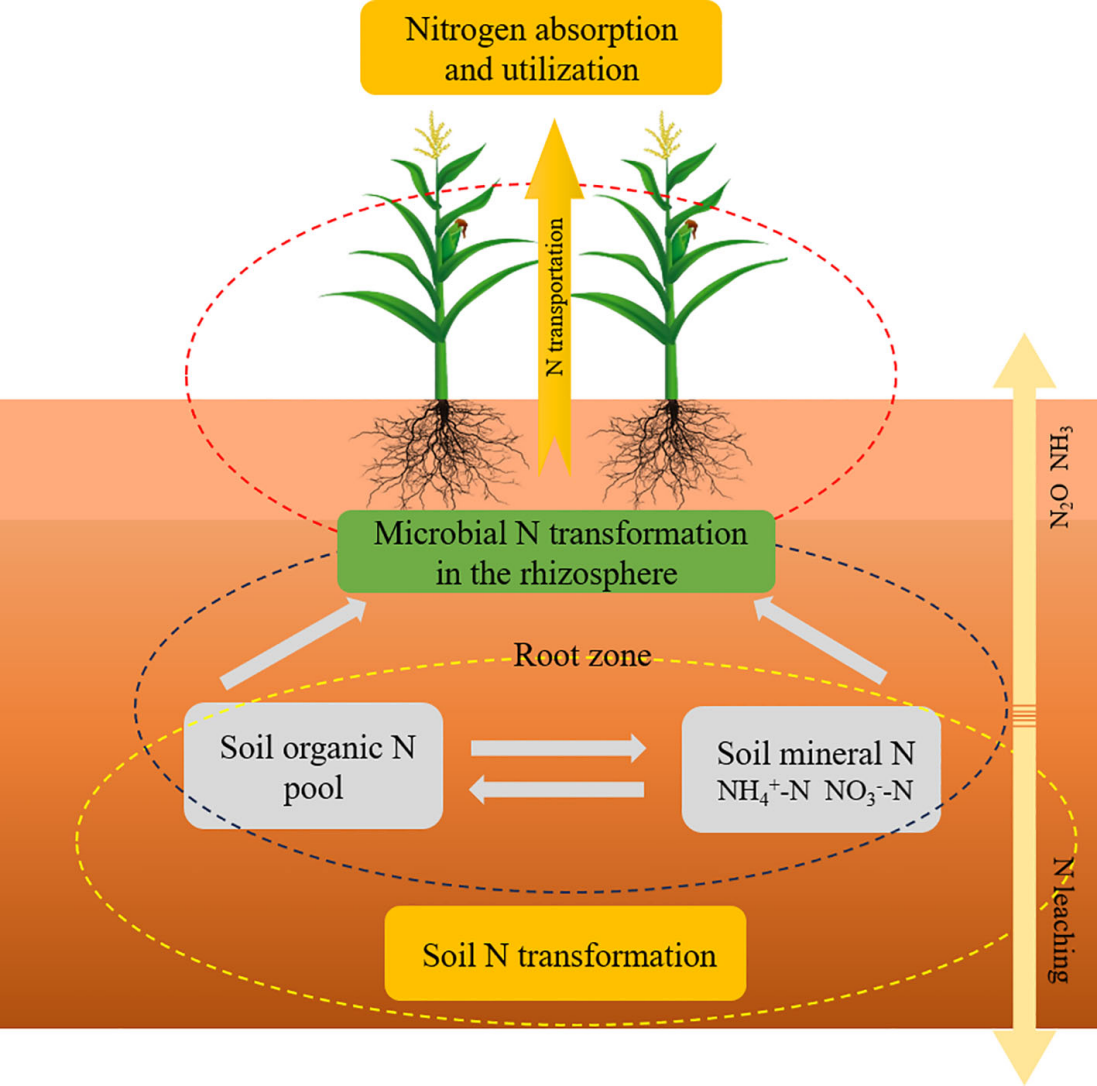
Relationship between soil nitrogen conversion and crop nitrogen uptake and utilization.

The nitrogen biogeochemical cycle involves processes such as nitrogen fixation, nitrification, denitrification, and ammonification, primarily mediated by soil microorganisms (Holz et al., 2023). Nitrogen exists in various forms in soil, classified as organic and inorganic nitrogen, with the organic content significantly surpassing the inorganic. Inorganic nitrogen is further divided into nitrate nitrogen and ammonium nitrogen based on its state (Cao et al., 2021). Nitrate nitrogen, a crucial nitrogen source for plants, is susceptible to losses through leaching and runoff because of its high mobility. Under anaerobic conditions, nitrate may be released through denitrification (Ju and Zhang, 2017). In specific scenarios, atmospheric nitrogen infiltrates the soil through fixation, transforming into inorganic nitrogen, which becomes accessible for plant uptake. This inorganic nitrogen produces compounds such as N_2_O, NO, and N_2_ by participating in NH_3_ volatilization, nitrification, and denitrification, these nitrogenous compounds then re-enter the atmosphere, finally completing the soil nitrogen cycle (**Figure 2**) (Jeffrey and Carla, 2004). The processes and transformations involved in the soil nitrogen cycle are orchestrated by soil enzymes and microorganisms (Jesper et al., 2006). Processes such as immobilization and mineralization are fundamental to the soil nitrogen cycle and have garnered considerable research attention.

### 2.1 Regulation of green manure application on soil nitrogen immobilization

Soil nitrogen immobilization plays a crucial role in supplying nitrogen to plants (Li Z. L. et al., 2021). Both leguminous and non-leguminous green manures, recognized for their nitrogen contributions, possess inherent abilities for nitrogen fixation. Leguminous green manures, characterized by substantial aboveground biomass, foster symbiotic interactions between their root systems and rhizobia, facilitating nitrogen fixation (Ben-Laouane et al., 2020). Upon incorporation into the field, these manures enhance soil nitrogen levels, improving nitrogen uptake and efficiency in primary crops (Coombs et al., 2017; Vincent-Caboud et al., 2019; Yang L. et al., 2019). A hectare of leguminous green manure can assimilate atmospheric nitrogen at rates ranging from 110 to 227 kg, potentially replacing or reducing the reliance on chemical nitrogen fertilizers (Mueller and Thorup-Kristensen, 2001; Xie et al., 2016b). According to the Food and Agriculture Organization estimates, global annual biological nitrogen fixation is approximately 130 million tons (Kakraliya et al., 2018). Leguminous plant-rhizobia symbiotic nitrogen fixation constitutes 65% − 70% of this figure, wherein the nitrogen fixed by leguminous plants can cater to 50% − 80% of the nitrogen requisites for plant development (Justes, 2018). Furthermore, both leguminous and non-leguminous green manures absorb inorganic nitrogen from soil, convert it into organic forms within the plants, and reintroduce it to the soil, contributing to soil nitrogen replenishment (Yang et al., 2022).

**Figure 3:**
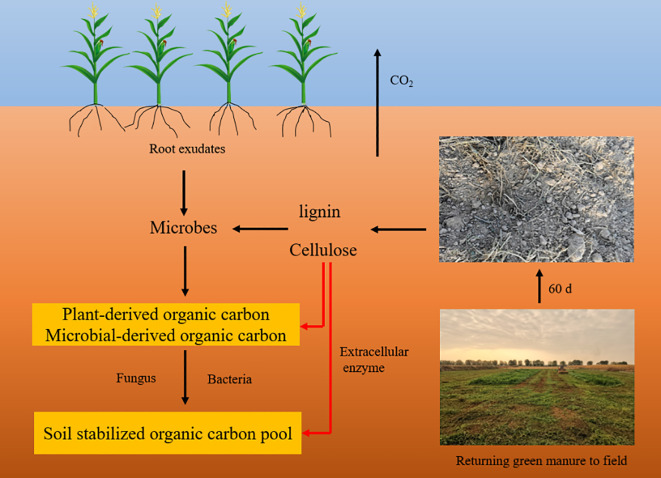
The production process of soil stable organic carbon pool after returning green manure to the field.

Soluble organic nitrogen and inorganic nitrogen are the most dynamic components in the soil matrix, available for direct uptake by plants and microorganisms or after specific transformations (Ji et al., 2018). Cultivating of green manure crops facilitates the absorption of liberated nitrogen, leading to soil nitrogen immobilization (Snapp et al., 2005). Introducing plant residues with a high C:N ratio into the soil enhances its nitrogen fixation potential (Vinther et al., 2004). Evidence suggests that soil organic carbon mass plays a pivotal role in driving soil nitrogen fixation (Cao et al., 2021). The cultivation and integration of green manures can elevate metrics such as microbial biomass carbon on the soil surface, labile organic carbon, dissolved organic carbon, and the content of resilient and tightly bound humus, thereby improving the quality of organic carbon (**Figure 3**) (Ye et al., 2015).

### 2.2 Regulation of green manure application on soil nitrogen mineralization

**Figure 4:**
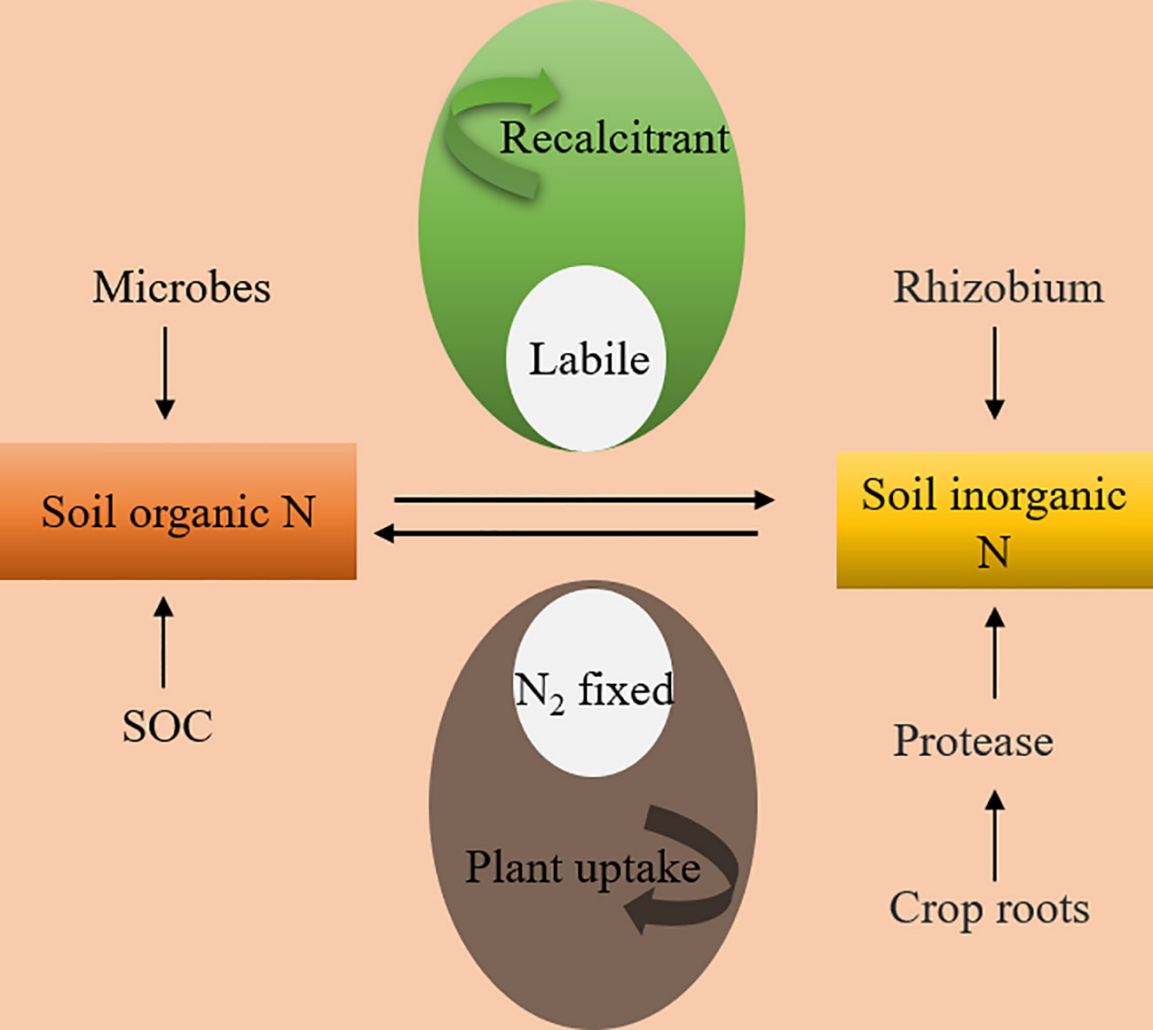
Nitrogen immobilization and mineralization process in farmland soil under green manure application.

Nitrogen mineralization is a key player in regulating soil-active nitrogen concentrations (Ouyang et al., 2008) (**Figure 4**). Upon the introduction of green manure, various organic nitrogen forms undergo transformation into inorganic nitrogen through mineralization. Some of these forms are absorbed by crops, while others are adsorbed by the soil. The remaining free inorganic nitrogen volatilizes as ammonia under alkaline conditions in drylands. (Fageria and Baligar, 2005) Conversely, in paddy fields, this nitrogen produces N_2_O, NO, and N_2_ through the denitrification process (Mccauley et al., 2012). Empirical evidence supports the idea that soil net nitrogen mineralization increases with the incorporation of green manures (Delgado and Follett, 2011). This enhancement primarily arises from green manure meeting the nitrogen requirements of soil microorganisms, catalyzing the activation and breakdown of bound-state mineral nutrients (Kuzyakov and Xu, 2013). Specifically, in terms of soil organic nitrogen, the decomposition of green manures provides energy and carbon reservoirs for microorganisms, invigorating the metabolic functions of ammonifiers and nitrifiers. Ammonifiers convert organic nitrogen into ammonia nitrogen, while nitrifiers oxidize this ammonia nitrogen into nitrate nitrogen, further augmenting inorganic nitrogen formation.

During the initial phase of green manure decomposition (approximately 20 days), easily decomposable components are rapidly mineralized by microorganisms, causing a significant increase in the soil*’*s inorganic nitrogen content (Carter et al., 2014). Simultaneously, an abundance of carbon sources reinforces the nitrogen immobilization effect orchestrated by microorganisms. In the intermediate and advanced stages of green manure decomposition (approximately 20 − 60 days), the depletion of carbon sources leads to a decrease in both mineralization and immobilization effects; however, a net positive growth trajectory persists (Liu et al., 2019). Various studies suggest that soil nitrogen mineralization is driven by microorganisms, with the stoichiometry of microbial biomass influencing the intensity of nitrogen mineralization (Li et al., 2019; Li et al., 2020). For example, during the decomposition of green manures, the rate depends on the soil C:N ratio (Jahanzad et al., 2016). A lower C:N ratio accelerates decomposition and nutrient liberation, while a higher ratio slows down the breakdown of green manures (Portugal et al., 2020). Therefore, choosing leguminous plants with a balanced carbon-nitrogen ratio or a mix of leguminous and non-leguminous species can balance soil nutrient sequestration and provision. An important aspect of green manure utilization is the synchronization of dynamic nutrient release with crop nutrient requirements (Brandsaeter et al., 2008; Kandel et al., 2018). Hairy vetch and rye are the predominant green manure crops in the Midwestern United States. Sievers and Cook (2017) reported that, compared with rye, the artificial cessation of Hairy vetch growth, results in an increased nitrogen release in terms of both quantity and rate. Approximately 30 days after halting Hairy vetch growth, nearly all contained nitrogen is liberated, coinciding with the peak nitrogen assimilation phase of 8-week-old maize. By contrast, rye*’*s nitrogen release spans a considerably longer duration, making it suitable for crops with minimal nitrogen demands. Therefore, under green manure application, aligning soil nitrogen mineralization and immobilization with crop nutrient demands can enhance efficient nitrogen absorption and utilization in subsequent crops.

### 2.3 Regulation of green manure application on soil nitrogen loss

#### 2.3.1 NH_3_ Volatilization

The presence of NH_3_ in soil originates from the ammonification of NH_4_^+^-N, catalyzed by urease (Farooq et al., 2022). Decomposition of green manures, whether leguminous or non-leguminous, results in increased concentrations of soluble nitrogen, promoting the ammonification of unstable nitrogen in the short term, and causing rapid initial fluctuations in NH_3_ volatilization (Janzen and Mcginn, 1991). While green manure decomposition enhances NH_3_ emissions, studies have shown that ammonia volatilization from shoot decomposition post - green manure application constitutes only 0.31% the total farmland ammonia volatilization (Ferrara et al., 2021). Chemical nitrogen fertilizers remain the primary source of ammonia volatilization, and incorporating leguminous green manure with these fertilizers amplifies soil ammonia losses (Zhang et al., 2022). Introducing leguminous green manure not only boosts the mineralization potential of organic nitrogen but also enhances ammonification and nitrification of ammonium nitrogen fertilizer, resulting in elevated NH_3_ emissions (Sun et al., 2021). Conversely, replacing a portion of nitrogen fertilizers with green manures can significantly reduce NH_3_ volatilization (Bai et al., 2015). Rana and Mastrorilli (1998) support this, demonstrating that approximately 70% of soil ammonia in green manure-treated farmlands is discharged within 2 days post chemical nitrogen fertilizer application. The NH_3_ flux regulation mechanism mirrors water vapor transport, and the decomposition of green manures releases abundant organic nutrients, such as organic carbon and nitrogen accelerating soil mineralization, reducing the interaction between ammonium nitrogen and soil colloids, increasing soil NH_4_^+^-N content, and catalyzing NH_3_ volatilization (Kulesza et al., 2022).

#### 2.3.2 N_2_O *e*mission

The application of leguminous green manure is widely acknowledged to increase N_2_O emissions in agricultural fields. Organic cultivation soils, compared to conventionally tilled soils, exhibit higher potential denitrification rates, increased organic matter content, and heightened microbial activity (Xie et al., 2016a). However, some research suggests that leguminous green manure might attenuate N_2_O emission intensity. Duan et al. (2019) propose an alternate perspective: the incorporation of leguminous green manure promotes nitrogen transformation within microorganisms, facilitating the conversion of N_2_O to N_2_, thereby reducing N_2_O production through soil nitrification and denitrification processes. This effect is supported by increased abundance of the ammonia-oxidizing bacterium AOB-amo A and the N_2_O-reducing gene nos Z. Adopting no-tillage practices and utilizing green manure plants with elevated C:N ratios can also reduce N_2_O emissions, attributed to improved soil aggregate structure stabilizing volatile nitrogen (Huang et al., 2004). Integrating non-leguminous green manure with chemical nitrogen fertilizers can enhances nitrogen retention in the soil, reduces N_2_O emissions from primary crop farmlands, and alleviates environmental concerns related to nitrate leaching or runoff. This is linked to a decrease in the quantity of nitrifying bacteria and a decrease in enzyme activity leading to decreased soil NO_3_^−^-N content (Momesso et al., 2022). A global meta-analysis suggests that fields cultivated with non-leguminous green manure exhibit the highest N_2_O emissions, primarily correlated with water-filled pore space (Kramer et al., 2006). In conclusion, discrepancies in N_2_O emissions depend on the specific applications of green manures and varying soil environmental conditions.

#### 2.3.3 NO_3_^−^-N leaching

NO_3_^−^-N, owing to its limited capacity for easy adsorption by anionic soil colloids, is the most mobile nutrient in the soil (Johnson and Cole, 1980). During rainy seasons, cultivating green manure crops can significantly mitigate nitrogen leaching, given their capacity to assimilate free mineral nitrogen from the soil (Guo et al., 2008; Basche et al., 2014). Some investigations indicate that non-leguminous cover crops have a more pronounced effect on reducing NO_3_^−^-N leaching compared with their leguminous counterparts. On average, non-leguminous cover crops reduce leaching by 70%, whereas leguminous cover crops achieve a reduction of only 23% (Zhao et al., 2020). Campbell et al. (2008) observed negligible nitrogen leaching in soils cultivated with leguminous crops, potentially attributed to regional soil texture variations.

A global-scale meta-analysis suggests that the cultivation and application of green manure crops in primary croplands markedly decrease nitrogen leaching (Abdalla et al., 2019). Bai et al. (2015) reported that Orychophragmus violaceus, when incorporated into maize fields, minimizes nitrogen migration in maize fields to deeper soil strata,virtually eliminating nitrogen leaching. Specifically, before the application of Orychophragmus violaceus, nitrate nitrogen in primary crop soils was distributed at depths of 0 − 180 *cm*, after the application of Orychophragmus violaceus, nitrate nitrogen remained predominantly within the tillage layer (Bai et al., 2015). Other studies support the idea that the growing leguminous and graminaceous species together helps balance nitrogen supply and reduce leaching (Frasier et al., 2017). Overall, green manure crops play a crucial role in reducing NO_3_^−^-N leaching and environmental contamination.

### 2.4 Response of soil microbial community characteristics to green manure application

Soil microbial communities are essential for nutrient cycling and maintaining soil ecological functions. The diversity and richness of these microbial communities serve as vital metrics for assessing the robustness of soil ecological functions (Yang X. M. et al., 2019). These microorganisms drive the material cycles of agricultural ecosystems through their involvement in soil organic matter mineralization, humus formation, decomposition, and nutrient transformation in plants (Putten et al., 2014). The degree of soil microbial development intrinsically dictates the transmutation of soil nutrients (Xu et al., 2020). Microbial decomposition is the predominant process that provides energy integral to the transformation of organic matter (Ma et al., 2023).

The introduction of green manure crops has a direct influence on soil bacterial communities and an indirect influence on these communities by altering soil characteristics (Zhang et al., 2017). Green manure application fosters enhancements in soil microbial biomass and enzyme activity, and the magnitude of these changes potentially correlates with the microbial species and quantity of the green manure used (Bowles et al., 2014; Chavarría et al., 2016). Leguminous green manures improve microbial community growth and boost soil nitrogen fixation, benefiting subsequent crops in various agricultural settings (Melander et al., 2020). After applying green manure, a notable surge in the relative abundance of Proteobacteria, Acidobacteria, Pseudomonas, and Nitrospira in the soil was observed (Gu et al., 2021). According to Ru et al. (2012), green manure, unlike inorganic fertilizers, markedly amplifies the relative abundance of Actinomycetes and Skermanella in the soil. Actinomyces, a predominant bacterial microflora ubiquitously distributed in alkaline soil, have branched mycelia that secrete hydrolases. These hydrolases break down insoluble organic substances in the soil, thereby enhancing organic mineralization, crop root development and enzyme activity, and crop nitrogen assimilation and utilization (Sanford, 2006). The introduction of green manures to fields also increases the population and vitality of arbuscular mycorrhizal fungi in the rhizosphere (Hontoria et al., 2019; Cruz et al., 2020). These fungi form symbiotic relationships with plant rhizospheres, facilitating nitrogen uptake and translocation in plants (Veresoglou et al., 2012).

Green manure application affects soil microbial communities modify the composition and diversity of soil microbial communities both directly and indirectly by enhancing the soil*’*s physical and chemical attributes (Yang et al., 2016). Specifically, introducing green manures to fields influences the soil microbial community structure by adjusting the sources of soil carbon and nitrogen (Xie et al., 2017). While microbial biomass carbon constitutes a minor fraction of the overall soil carbon, it mirrors the microbial utilization of carbon sources for growth and reproduction, offering energy crucial for crop nutrient transformation (Pelz et al., 2005). Organic fertilizers with high C:N ratios contain low-molecular-weight organic compounds, which foster the formation of loose, porous soil aggregates favorable for microbial growth and soil nitrogen fixation (Van Zwieten et al., 2014). The degree of change in bacterial functions depends on the bacterial species and quantity of green manures applied (Mbuthia et al., 2015). Soil microbial community structure after green manure application is predominantly influenced by various factors including soil soluble organic carbon, NO_3_^−^-N, and microbial biomass (Khan et al., 2019). This underscores that in the context of green manure deployment, the characteristics of soil microbial communities are molded by an array of determinants, including the nature and attributes of green manures, organic matter input, and soil physicochemical properties. These factors synergistically determine the composition, diversity, and functions of soil microbial communities.

## 3 The high-efficient nitrogen absorption and utilization mechanism of main crops under green manure application

The absorption and utilization of nitrogen in crops is a multifaceted biological and ecological process, involving nitrogen uptake within the crop rhizosphere; internal transport and transformation of nitrogen; the distribution, synthesis, and metabolism of nitrogen within the plants (Souad Insaf et al., 2021). This intricate mechanism involves various aspects such as plant physiology, genetic modulation, rhizosphere microbial interactions, and abiotic environmental determinants of the soil. The efficiency of nitrogen utilization in plants depends not only on soil nitrogen availability and uptake efficiency but also on nitrogen availability from temporary reservoirs such as proteins in source leaves, stems, or roots and the efficiency of amino acid transport (Zhu et al., 2014). Contemporary studies on the influence of exogenous organic matter on nitrogen absorption and utilization in primary crops predominantly focus on nitrogen uptake efficiency, root morphology, and nitrogen absorption and distribution.

### 3.1 Promotion of soil nitrogen transformation and soil nitrogen availability

The balance between mineralization and immobilization governs the dynamics and availability of soil mineral nitrogen, significantly affecting nitrogen availability in agricultural soils and its subsequent absorption and utilization by crops (Vitousek et al., 2010). After adding green manures to fields affects soil organic nitrogen components: soil microbial biomass nitrogen and mineral nitrogen show opposite trends at different stages of primary crop growth. This indicates that green manures adjust the soil nitrogen fixation-mineralization balance to meet the nitrogen needs of the primary crops (Goyal et al., 1993). When crops need nitrogen, soils abundantly provide available nitrogen. By contrast, when the crop nitrogen demand is low, inorganic nitrogen is converted to organic nitrogen in the soil. This mechanism not only reduces gaseous and nitrate losses but also improves soil quality (Robertson and Vitousek, 2009). Nitrogen reduction trials have confirmed that the addition of green manure for improved nitrogen management in agriculture increases nitrogen absorption and utilization efficiency, without reducing crop yield, when compared with conventional management practices (Ding et al., 2018). Additionally, this approach offers environmental benefits (Bai et al., 2015): first, the reduced environmental losses (such as N_2_O emissions, NH_3_ volatilization, and leaching) compensate for the lower soil nitrogen input; second, reintroducing green manure to the soil improves crop nitrogen uptake by enhancing the soil environment.

### 3.2 Promote crop yield and nitrogen uptake

In both arid regions and paddy fields, the strategic application and deployment of various green manures can significantly improve nitrogen uptake and utilization efficiency, ultimately enhancing the yield of subsequent crops (Silva et al., 2020). Long-term cultivation and incorporation of green manure have demonstrated the potential to elevate nitrogen uptake by primary crops while reducing nitrogen losses (Liang et al., 2022). Spectral image analysis conducted by Wittwer and Heijden (2020) showed that the application of leguminous green manure increased maize nitrogen absorption by 79 kg·ha^−1^. Notably, leguminous green manure crops present a promising alternative to certain chemical nitrogen fertilizers, supplementing nitrogen availability, promoting enhanced nitrogen uptake, and stimulating growth in subsequent crop stages (Abera and Gerkabo, 2021). In situations where nitrogen is reduced by 30%, compared to the sole use of chemical nitrogen fertilizers, the incorporation of non-leguminous green manure, specifically orychophragmus violaceus, resulted in a significant 9.9% and 10.2% increase in rotation maize yield and biomass, respectively, upon incorporation. Simultaneously, there was a remarkable 26.7% improvement in nitrogen utilization efficiency, highlighting the symbiotic relationship between green manure application and soil nitrogen concerning crop nitrogen uptake and utilization (Bai et al., 2015). However, disparities exist in the impact of green manure types and incorporation methods on crop yield and nitrogen dynamics. In arid irrigated regions, particularly within the vicia sativa-corn rotational framework post-wheat harvest, the consistent incorporation of green manure and residues has been shown to elevate both wheat and corn yields alongside nitrogen utilization efficiency (Lyu et al., 2020b). Notably, full green manure application yields superior outcomes (Lyu et al., 2020a). Conversely, in rainfed agricultural zones during water-deficient periods, cultivating leguminous green manure during summer fallow intervals may lead to a reduction in subsequent winter wheat yield due to green manure-induced soil moisture consumption (Han et al., 2017). However, no such yield reduction was observed upon nitrogen decrement, ensuring an enhancement in nitrogen utilization efficiency (Zhang et al., 2015). Naveen et al. (2020), however, documented a yield deficit in Pennsylvania when green manure crops were introduced during the V2 phase of corn development. This finding suggests that the optimal utility of green manure crops lies in temperate regions with copious rainfall. Danga*’*s investigation in humid locales revealed that leguminous crops, when rotated with cereals, did not compromise the yield of subsequent crops despite their consumption of soil moisture (Danga et al., 2009). In summary, customizing the application patterns of green manure to the specificities of various agricultural contexts can optimize crop nitrogen uptake and utilization efficiency, yield, and plant nitrogen absorption.

### 3.3 Promote crop nitrogen transport and distribution

**Figure 5:**
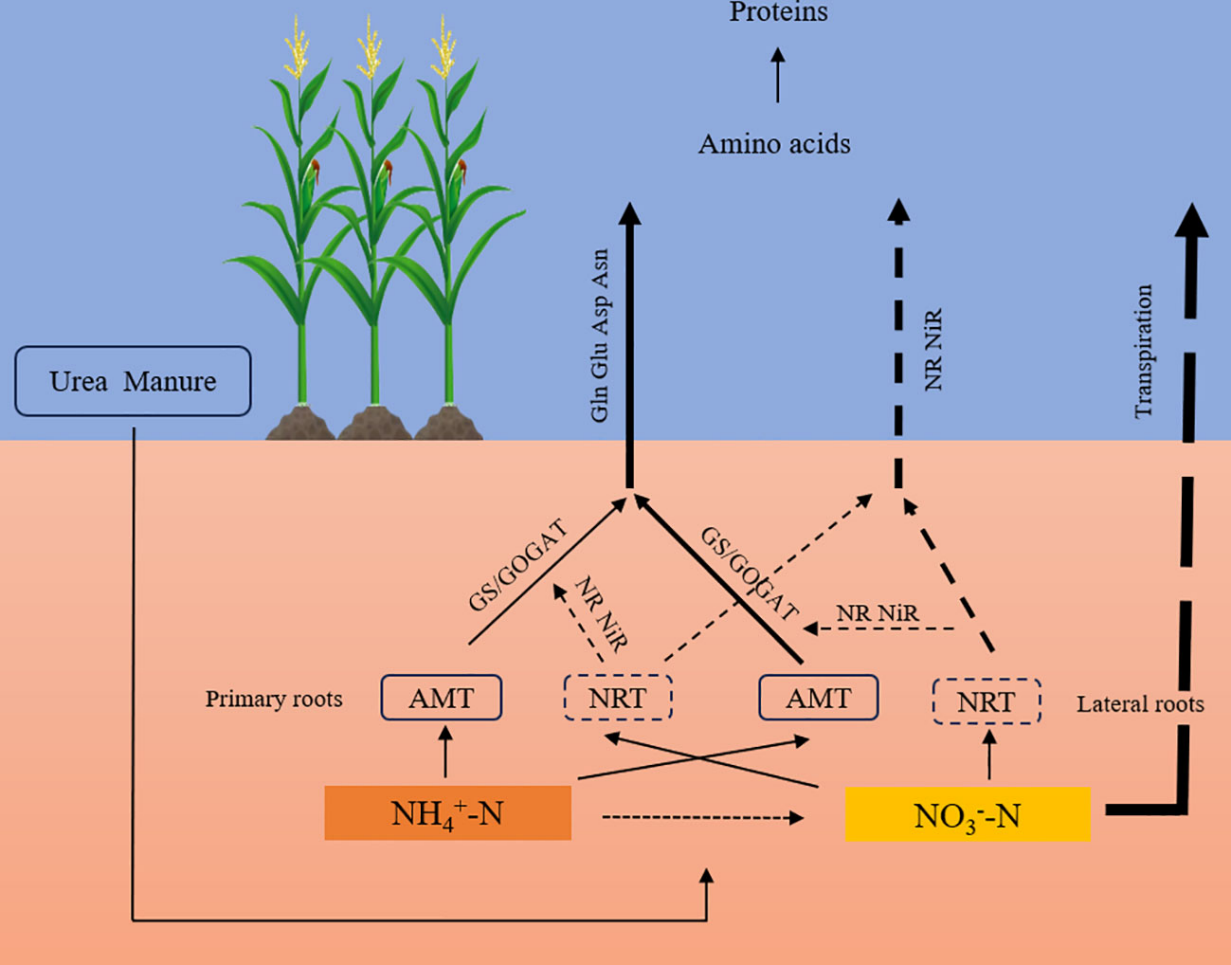
Plant nitrogen absorption and transport process.

Nitrogen is utilized by plants through absorption, transportation, and assimilation (Mokhele et al., 2012). During the vegetative growth phase, the primary nitrogen reservoirs are roots and leaves, whereas during the reproductive phase, the primary nitrogen reservoirs are flowers, fruits, and seeds (Robe and Griffiths, 2010). In roots, nitrate absorption from the soil occurs through nitrate transport proteins (NRTs), and ammonium absorption from the soil occurs through ammonium transport proteins (AMTs) (Luo et al., 2013). After absorption, nitrogen is transported from the root to the shoots through the xylem, and within the phloem, it migrates from source leaves to sinks. After the application of ammonium-based fertilizers, most of the absorbed ammonium salts are integrated into organic compounds within the plant roots, simultaneously releasing an equivalent quantity of H^+^; these H^+^ reduce the pH of the surrounding soil environment, causing localized soil acidity (Raven and Smith, 1976). Some of the nitrate ions are either transported within the xylem or stored in the vacuoles of root, stem, and other storage cells, which modulate plant ion balance and osmotic pressure (Müller et al., 2004). A smaller fraction undergoes reduction to ammonia via nitrate reductase (NR) and nitrite reductase (NiR), feeding into amino acid synthesis pathways and leading to glutamine production. With the influence of transpiration, the majority of NO_3_^−^-N is channeled via the xylem to the aboveground components of the plant. Glutamine synthase (GS), a pivotal enzyme in nitrogen assimilation, acts in conjunction with glutamate synthase (GOGAT) to convert ammonium to glutamine, and further releasing glutamate and aspartate. In essence, nitrate nitrogen is integrated into essential amino acids for assimilation by crops, a process catalyzed by nitrogen-transforming enzymes(see **Figure 5**).

Recent studies have made significant progress in understanding nitrogen transport, metabolism, and regulatory mechanisms. However, there is limited research on the influence of green manure affects nitrogen transport and distribution. For the pea plant (Pisum sativum), increasing amino acid accumulation and transport in the phloem can improve nitrogen absorption by roots, affecting the assimilation of available nitrogen in various plant parts such as source and sink regions (Kulesza et al., 2022). Lyu et al. (2020a) documented that full addition of leguminous green manure, as opposed to just stubble, enhances nitrogen movement from leaves to the grains of primary crops, a phenomenon also noted with non-leguminous green manures (Li T. Y. et al., 2021). Soil and plant enzymes, especially GS and nitrate reductase, play a vital role in improving crop nutrient transport, marking grain protein synthesis (Li T. Y. et al., 2021). Green manure boosts soil enzyme activities related to nitrogen absorption and use, as well as these enzymes in leaves, aiding grain protein formation and nutrient content (Fu et al., 2022). Nitrogen from decomposed green manures in soil forms a significant part of the nitrogen uptake in primary crops. In leguminous-gramineae crop rotations, 34% of gramineae crops’ nitrogen uptake comes from nitrogen introduced into the soil by preceding leguminous crops (Laberge et al., 2011). Li et al. (2015) conducted field experiments in Brazil using the ^15^N labeling technique and reported that the recovery rate of ^15^N in corn kernels after the incorporation of vicia villosa varied between 9.8% and 10.1%, depending on the extent of vicia villosa cover.

### 3.4 Promote crop root development and improve rhizosphere environment

The ability of roots to absorb and transport soil nitrogen is crucial for plant nitrogen efficiency. Root growth and development determine the soil area and nutrient range accessible to plants (Fageria and Moreira, 2011). Root exudates alter the rhizosphere, affecting soil nitrogen availability (El-Shatnawi and Makhadmeh, 2001). Research on green manure and crop roots mainly focuses on the morphological attributes of the root system. There is a consensus that reintroducing green manures to fields boosts the growth of primary crop roots, enhances nutrient uptake, and increases root biomass and the root-to-shoot ratio. These benefits are linked to improved soil physicochemical properties due to green manure application (Mandal et al., 2003; Kandel et al., 2018). The interaction between mature leguminous green manure and soil microorganisms accelerates mycelial growth in the soil and decomposition of extracellular polysaccharides secreted by various microorganisms. This interaction leads to an increased production of organic acids and other root exudates, which, combined with soil aggregates, mycelia, and minerals, create a nutrient-rich, well-structured rhizosphere environment for the crop (Van Zwieten et al., 2014).

**Figure 6:**
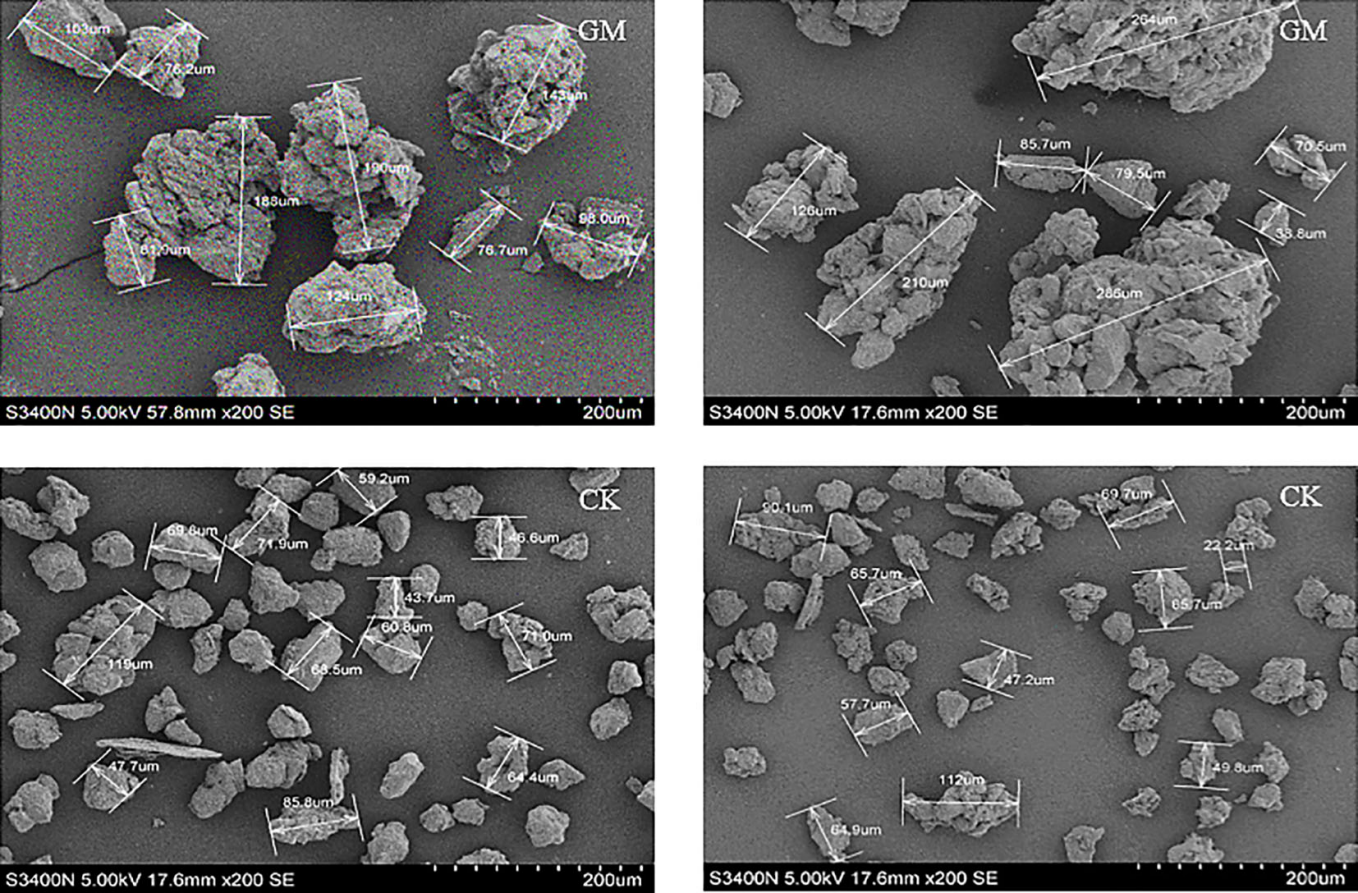
Characteristics of soil aggregate under green manure application. GM is green manure application, CK is no green manure application. The published images above are the results of research by the first author of this article and have been authorized by its publication journal and all authors.

The rhizosphere environment influences nitrogen form and availability, affecting crop nitrogen absorption and utilization (Moreau et al., 2019). Introducing exogenous organic matter such as green manure significantly affects the soil-plant nitrogen transformation. Green manure application enhances nutrient cycling by improving soil qualities such as water-holding capacity, porosity, aggregate density, and microbial population dynamics and vigor (Letter et al., 2003; Haruna and Nkongolo, 2015). Soil moisture, temperature, and humidity are key factors influencing nitrogen absorption by the plant and transport within the plant parts (Budhar and Palaniappan, 1996). The composition and stability of soil aggregates affect soil material exchange and energy equilibrium. Notably, water-stable aggregates of size 1–10 mm are an optimal substrate for crop nutrient uptake (Tisdall and Oades, 1982; Jastrow, 1996). Lyu et al. (2022) performed a scanning electron microscopic analysis in their study and reported that, compared with just stubble application, full green manure incorporation improves soil aggregate microstructure and increases the presence of larger aggregates (**Figure 6**).

## 4 Frontier technologies and methods for exploring crop nitrogen transformation under green manure application

### 4.1 ^15^N isotope tracer technology

Global agricultural scientists employ various methods to study the effects of leguminous crops. One such method is the ^15^N labeling technique, combined with numerical models, to assess nitrogen transformation efficiency (Mary et al., 1998; Van Zwieten et al., 2014). This non-invasive method enables the contribution and distribution of different nitrogen sources in nitrogen transformation and the exchange mechanism of nitrogen between different soil components. The ^15^N tracer technology has been vindicated to be an effective tool for investigating nitrogen mineralization and fixation, nitrogen loss means, and the relationship between nitrogen forms and plant availability (Jin-bo et al., 2013). In the late 1930s, Rittenberg et al. (1939) first applied the ^15^N tracer technology to study biological nitrogen fixation. Mccauley et al. (2012) utilized the ^15^N natural abundance method to determine the δ ^15^N of leguminous crops at various planting times, aiding farmers in selecting suitable leguminous crops for different seasons and ecological areas to optimize farmland planting systems. In China, the application of ^15^N tracing technology focuses on nitrogen absorption, utilization, transport, and distribution in crops. (Junjun et al., 2019) examined the ^15^N isotope composition in N_2_O molecules to determine the relative contributions of denitrification and nitrification. Zhu et al. (2014) used ^15^N labeling to study nitrogen absorption and utilization in rice following the application of Chinese milk vetch to the field. Currently, stable isotope labeling technology is primarily used to explore the nutrient flow status in soil-plant systems in agriculture, but research on the metabolic links in plants and the flow status of the whole ecosystem. For example, what is the metabolism status of green manure after it is used as forage grass in animal husbandry system?

### 4.2 Genomics: high-throughput sequencing technology

Since the 21st century, advancements in genomics, transcriptomics, and high-throughput sequencing technology and the development of bioinformatics have significantly propelled soil microbiology research. Past studies have applied genomics to explore the genomic resources of green manure crops and understand the gene expression mechanisms, metabolic pathways, and secondary metabolites (Sathyanarayana et al., 2017). High-throughput sequencing technology, for example, has been used to examine soil bacterial communities’ characteristics and functional diversity. (Liu et al., 2019) used high-throughput sequencing technology to investigate the spatial distribution patterns of functional microorganisms (AOA and AOB) involved in nitrogen transformation in the soil. They discovered that AOA abundance correlated positively with soil carbon content, while AOB abundance correlated significantly positively with soil pH. These methods offer deep insights into soil microbial community structures, functional activation of soil microorganisms under green manure application, and the role of microorganisms in soil nitrogen transformation and the regulatory mechanism underlying the gene expression of key enzymes.

### 4.3 Infrared spectroscopy and ^13^C nuclear magnetic resonance technology

Infrared spectroscopy and ^13^C nuclear magnetic resonance are can be used to study the decomposition and nutrient release of green manures. Near-infrared spectroscopy and infrared spectroscopy enable the non-destructive determination of nitrogen in soil and plant samples. These efficient and rapid detection technologies facilitate large-scale research on crop nitrogen transformationWittwer and Heijden (2020) adopted spectral image analysis showed that leguminous green manure crops can compensate for nitrogen availability, have improved nitrogen absorption, and show accelerated crop growth in later stages. The ^13^C nuclear magnetic resonance method simultaneously monitors the distribution and transformation of carbon and nitrogen in the soil, revealing the effect of carbon-nitrogen interaction on soil nitrogen transformation. It is conducive to deeply understand the coupled cycle of soil carbon-nitrogen and promote the development of ecological agriculture. Additionally, the integration of system dynamics models, ecological models, and nitrogen cycle models helps perform numerical simulation and prediction of crop nitrogen transformation after green manure application. These models are effective because they comprehensively consider the influence of multiple factors to predict the effects of different management measures on nitrogen transformation.

## 5 Prospects for the regulation of soil nitrogen transformation and nitrogen absorption and utilization in crops by green manure application

Amidst global agricultural development, intensified by population growth, challenges include declining soil fertility and wastage of nitrogen resources have emerged as pressing agricultural concerns. Green manure application is an eco-friendly agronomic option, but ongoing research has revealed both existing challenges regarding green manure application and promising future opportunities. First, the high cost of green manure seeds, together with the economic inputs for sowing, incorporation, and other procedures, increases production costs. Second, despite numerous green manure varieties, only few are multi-resistant and suitable for cultivation in diverse regions. Third, how to incorporate green manure into cropping systems according to the characteristics of different ecoregions and resolve the contradictions between green manure and cash crops is a major issue for green manure cultivation and utilization. Unreasonable cultivation leads to competition for water and nutrients between green manure and cash crops, hindering the normal growth of cash crops and severely limiting the contribution of green manure to modern agriculture. Fourth, while increasing soil organic carbon content and improving soil quality and fertilizer efficiency through green manure incorporation, large amounts of greenhouse gas emissions are also generated. How to optimize cultivation methods or incorporation modes to reduce greenhouse gas emissions remains a challenge. Fifth, the fertilizer effect of green manure is slow. Not replenishing chemical fertilizers in the short term will lead to reduced yields of cash crops. Future research should focus on optimizing soil nitrogen dynamics and crop nitrogen uptake through green manure incorporation.

### 5.1 Deepening insights into the ecological impact of green manure application

The regulatory dynamics between green manure and soil nitrogen conversion and the crucial role of soil microorganisms in nitrogen cycling warrant in-depth exploration. Understanding the mechanisms by which nitrogen is lost through microbial processes can help reduce emissions. Knowledge of these microbial interactions under the influence of green manure can improve application methods and increase efficiency in using natural resources.

### 5.2 Green manure selection and distribution optimization

Different green manure crops affect soil nitrogen dynamics and crop nitrogen assimilation in various ways; therefore, choosing and arranging them wisely is important. Future research should be conducted based on the judicious selection and arrangement of green manures. Future investigations should elucidate the soil-enhancing effects of different green manures in various regions, probe the repercussions of varied green manures on soil properties under distinct planting paradigms—such as monoculture, intercropping, and mixed sowing—and create a detailed database of their soil improvement qualities of various green manures. This will provide farmers with clear guidelines to improve nitrogen efficiency and reduce nitrogen wastage.

### 5.3 Advancement in soil nutrient cycling and farmland ecosystem service functions

Green manure incorporation not only affects soil nitrogen dynamics and crop nitrogen availability but also interacts with other soil nutrients and ecosystem services. Future studies should consider nitrogen cycles along with other nutrient cycles, explore the effects of green manure application on soil health and ecosystem functions, and enhance the role of green manure in fostering sustainable agriculture.

## 6 Conclusions

Green manure crops, as environmentally friendly nitrogen sources, are cultivated in various regions globally. Both leguminous and non-leguminous varieties play a key role as intrinsic catalysts in soil nitrogen cycling. A salient characteristic of green manure application lies in its ability to maintain a balance between nitrogen fixation and mineralization, preserving soil health and providing essential nitrogen to grain crops with requisite nitrogen. Integrating green manures with chemical nitrogen fertilizers can reduce gaseous emissions of nitrogen and prevent nitrate leaching. Green manure deployment also improves the composition and functionality of soil microbial communities. Incorporating green manures into agricultural systems augments the nitrogen assimilation efficacy in primary crops in terms of crop nitrogen uptake and efficiency, evidently increasing crop yields, improving plant nitrogen uptake, facilitating nitrogen transport and distribution, enhancing root development, and regulating rhizosphere attributes such as soil aggregates. Future research could use advanced techniques such as molecular ecology to uncover the ecological mechanisms underpinning green manure application, thus enhancing farmland ecosystem services.

## Author contributions

HL: Writing – original draft, Conceptualization. YL: Formal Analysis, Writing – review & editing. YW: Visualization, Writing – original draft. PW: Visualization, Writing – original draft. YS: Supervision, Writing – original draft. XY: Conceptualization, Writing – review & editing. FW: Methodology, Writing – review & editing. AY: Methodology, Writing – review & editing.
